# Regulation of left atrial fibrosis induced by mitral regurgitation by SIRT1

**DOI:** 10.1038/s41598-020-64308-6

**Published:** 2020-04-29

**Authors:** Dong Zhang, Bo Li, Bin Li, Yue Tang

**Affiliations:** 1 Beijing Jishuitan Hospital, Department of Thoracic Surgery, Beijing, China; 2 The Seventh Affiliated Hospital, Sun Yat-sen University, Department of Cardiac Surgery, Shenzhen, China; 3 Animal Experimental Centre, Beijing Key Laboratory of Preclinical Research and Evaluation for Cardiovascular Implant Materials, State Key Laboratory of Cardiovascular Disease, Fuwai Hospital, National Centre for Cardiovascular Disease, Chinese Academy of Medical Sciences and Peking Union Medical College, Beijing, China

**Keywords:** Target validation, Cardiac hypertrophy

## Abstract

SIRT1 (silent information regulator 1) is a histone deacetylase. It can sense the energy level in cells and delay cell senescence, leading to resistance to external stress and improving metabolism. Mitral regurgitation (MR) is a common disease in cardiac surgery. However, there are no previous studies on SIRT1 and left atrial fibrosis caused by MR. In this study, we aimed to explore the regulatory effect of SIRT1 on left atrial fibrosis induced by MR. We used Guizhou miniature pigs to establish an MR model and a sham operation model after anaesthesia induction and respiratory intubation, and these model animals were followed for 30 months after the surgery. The differential distribution and expression of SIRT1 and collagen I in the left atrium was determined by immunofluorescence and Western blotting. Furthermore, we treated NIH3T3 fibroblasts (CFs) with resveratrol and Angiotensin II (Ang II) to analyse the specific mechanism involved in the development of myocardial fibrosis. The results showed that the MR model was successfully constructed. There were 8 pigs in the MR group and 6 pigs in the control group. In both the animal experiments and the cell experiments, the expression of collagen I in the MR group was increased significantly compared to that in the control group, while the expression of SIRT1 was decreased.

## Introduction

Atrial remodelling (AR) refers to a series of changes in size, including shape, wall thickness and tissue structure, due to increased or damaged load of the atrium. This pathophysiological process involves lesion repair and compensation of the atrium^1–3^. AR is characterized by atrial dilation, interstitial fibrosis, and an abnormal effective refractory period (ERP). AR can impair and eliminate atrial function, such as reserve function in ventricular systole and auxiliary pump function in late diastole, and even cause serious complications^4–6^.

The primary histological characteristic of AR is atrial fibrosis (AF)^7–10^. AF is usually caused by excessive fibrinogen deposition due to the dysregulation of extracellular matrix metabolism after atrial myocyte damage. AF plays an important role in the mechanism of atrial fibrillation and is the recognized pathological basis of atrial fibrillation. Atrial fibrosis is an irreversible pathological change inatrial tissue and is also the pathological basis of persistent atrial fibrillation, left atrial stiffness syndrome^11–13^ and other diseases. Recently, the development of antifibrotic drugs has been the focus of many studies, but there are few existing multitarget antifibrotic drugs, and they have poor long-term efficacy in heart diseases.

In an earlier study, we integrated and analysed the pathways related to fibrosis through the Genespring Platform of Agilent Technologies and found that SIRT1 (silent information regulator 1), a gene silencing regulatory protein, is the core protein that regulates many fibrosis-related pathways in multiple organs^14–16^.

SIRT1 is a highly conserved nuclear protein that is widely expressed in various tissues in the body. After activation, SIRT1 enters the nucleus from the cytoplasm and regulates the activity of nuclear transcription factors^17–19^. SIRT1 not only deacetylates histones to regulate gene transcription but also deacetylates some nonhistones, such as FOXOs (FOXO family of fork protein transcription factors), p53 (tumour suppressor factor), MyoD (myogenic determinant factor), PPAR-gamma (peroxidase-activated receptor), and PGC alpha (peroxidase-activated receptor costimulating factor alpha)^20,21^, to regulate cell proliferation, differentiation, energy metabolism, oxidative stress, ageing, apoptosis, and other pathological physiological processes^22,23^. Thus far, studies on SIRT1 have mostly focused on mice, rats and other small animal models. Since small animals are prone to heart failure and cardiac dysfunction after modelling, long-term feeding is difficult; thus, there is a lack of long-term observational studies on SIRT1 and myocardial fibrosis simulating the clinical disease process.

To preliminarily explore the role of SIRT1 in left atrial remodeling and myocardial fibrosis, this experiment aimed to establish a mitral regurgitation (MR) model in miniature pigs and carried out long-term observations on the regulatory role of SIRT1 in mitral regurgitation-induced left atrial fibrosis.

In a previous experiment, we successfully established a mitral regurgitation model by using a homemade “built-in heart valve cutter”. These model animals were followed for 30 months after the surgery. Enlargement and fibrosis of the left atrium were significant greater in the experimental group than the control group, and left atrial systolic function decreased significantly^24^. Based on previous studies, we aimed to explore the regulatory effect of SIRT1 on left atrial fibrosis induced by MR.

## Materials and Methods

### Animals and groups

Our experiments were conducted on Guizhou miniature pigs (10 months old, 25–35 kg) in China. The animals were obtained from the animal centre at the Fuwai Hospital of Peking Union Medical College (Beijing, China). The animals were maintained under standard conditions, including a temperature of 20–25 °C, a relative humidity of 50–70%, a ventilation frequency of 8–10 times/h, a 12-h light/dark cycle and adequate water and food. All the experimental protocols were approved by the Ethical Committee of the Fuwai Hospital of Peking Union Medical College. All methods in this study were performed in accordance with the relevant guidelines and regulations.

The experimental animal groups included the model group (mitral regurgitation group, 11 pigs) and the control group (sham operation group, 6 pigs).

The observation time of all animals was 30 months after the operation.

The criteria for establishing the model of mitral regurgitation and the inclusion criteria of the research group were based on previous relevant studies^25^, and severe mitral regurgitation was selected as the inclusion criteria.

### Histological analyses

The left atrium was removed from the heart and fixed in 10% formal in for 48 h. Specimens measuring approximately 1.0 cm × 0.5 cm were obtained from the left posterior wall of the left atrium central and left ventricular anterior wall. Epicardial connective tissue was removed to avoid overestimating the degree of fibrosis. Sirius Red staining was performed using a kit from Leagene (Beijing, China) according to the manufacturer’s protocol. Collagen I was stained red and orange, collagen III was stained green, and cell nuclei were stained blue. The degree of myocardial fibrosis was measured and quantified by Sirius Red staining using Image-Pro Plus 6.0. For each specimen, 10 fields were randomly selected, and the proportion of fibrotic areas was statistically analysed. The collagen volume fraction (CVF) was calculated as follows: CVF (%) = (total area of collagen/total area of the image) × 100%.

### Immunofluorescence

Cardiac tissues from the different groups were prepared as frozen sections for immunofluorescence analysis. Frozen sections (4.5 μm) and cardiac fibroblasts were fixed for 20 min in 4% paraformaldehyde (PFA) at room temperature. After fixation, they were blocked with 5% bovine serum albumin (BSA) in Tris-buffered saline with Tween (TBST) for 1 h, incubated with appropriate primary antibodies at 4 °C overnight and incubated with secondary antibodies (Abcam, UK) for 1 h at room temperature. Then, the sections and cells were stained with 1.5 μM 2-(4-amidinophenyl)-6-indolecarbamidine dihydrochloride (DAPI; Sigma, St. Louis, Missouri, USA) for 10 min. Analysis of the images of the frozen sections was performed using a software programme (Olympus, Japan). Protein localization in the cells was observed and captured with a laser scanning confocal microscope (LSM5, Zeiss, Jena, Thuringia, Germany).

### Cell culture and intervention

NIH3T3 mouse embryonic fibroblasts (CFs) were cultured in DMEM (Gibco, USA) containing 10% foetal bovine serum (FBS) (Gibco, USA), penicillin and streptomycin in an incubator with a 5% CO_2_ atmosphere at 37 °C. All the CFs used in the experiments were from the second to the fourth generations after isolation.

Resveratrol (a SIRT1 agonist) interferes with NIH3T3 fibroblasts. The cells were divided into two groups: the control group (cultured with normal DMEM) and the resveratrol group. In the early stage of the experiment, we applied different concentrations of resveratrol (0, 20, 40, 80, 160 μmol/L) to the NIH3T3 fibroblasts for 36 h. The MTT method was used to detect cellular activity, and the optimum concentration of resveratrol was 80 μmol/L. That is, at 80 μmol/L resveratrol, the activity of the NIH3T3 myocardial fibroblasts showed the most significant inhibition. According to the above experimental results, the optimal concentration of resveratrol (80 μmol/L) was selected to treat the NIH3T3 cells for 36 h, and the proliferation of fibroblasts was observed under a microscope.

Angiotensin II (Ang II) interferes with NIH3T3 fibroblasts. The cells were divided into two groups: the control group (cultured with normal DMEM) and the Ang II group. Cellular proteins were extracted after 0 h, 6 h, 12 h, 24 h, 36 h and 48 h by Ang II stimulation. Western blotting was used to analyse the expression of SIRT1 and collagen I in all groups at each time point.

### MTT assay for cell viability

Cell viability was evaluated using a colorimetric method based on the metabolic reduction of 3-(4,5-dimethylthiazol-2-yl)-2,5-diphenyltetrazolium bromide (MTT) dye to formazan, as previously described^26^. Briefly, cardiac fibroblasts were plated onto 96-well plates at a density of 8,000 cells per well. After another 24 h, the cells cultured at each time point were rinsed with phosphate-buffered saline (PBS), and then, MTT was added. Next, 4 h later, dimethyl sulfoxide (DMSO, Sigma-Aldrich, Inc., USA) was added to reduce the resulting formazan, and the cells were incubated for 15 min at 37 °C. Finally, the absorbance of each solution was measured at 570 nm.

### Western blot analysis

Proteins were extracted from the CFs and the cardiac tissues with RIPA buffer and collected via centrifugation at 12000 × g. Then, the proteins were separated using SDS-PAGE and transferred to PVDF membranes. The membranes were blocked and incubated with primary antibodies at 4 °C overnight. Antibodies against GAPDH (1: 1000, Golden Bridge, China), collagen I (1: 1000, Abcam, UK) and SIRT1 (1: 1000, Abcam, UK) were used. After incubation with secondary antibodies, the membranes were scanned directly using an ultraviolet imaging system.

### Statistical analysis

All values were analysed with SPSS 24.0 and are expressed as the mean ± SE of the mean. A single sample t-test was used to compare the mean of the samples with the known population mean. Independent samples t-tests were used to compare two sample means. Two-way ANOVA with repeated measures was conducted to examine differences between the groups and between measurement times. Independent samples t-tests were used for intergroup comparisons, and paired samples t-tests were used for intragroup comparisons. The χ^2^ test was used to examine the statistical significance of the positive rate in the atrial fibrillation susceptibility test between the two groups. P-values less than 0.05 were considered significant. GraphPad Prism 7 software was used for the statistical analyses.

## Results

### Pathological results of left atrial structural remodelling

**Figure 1:**
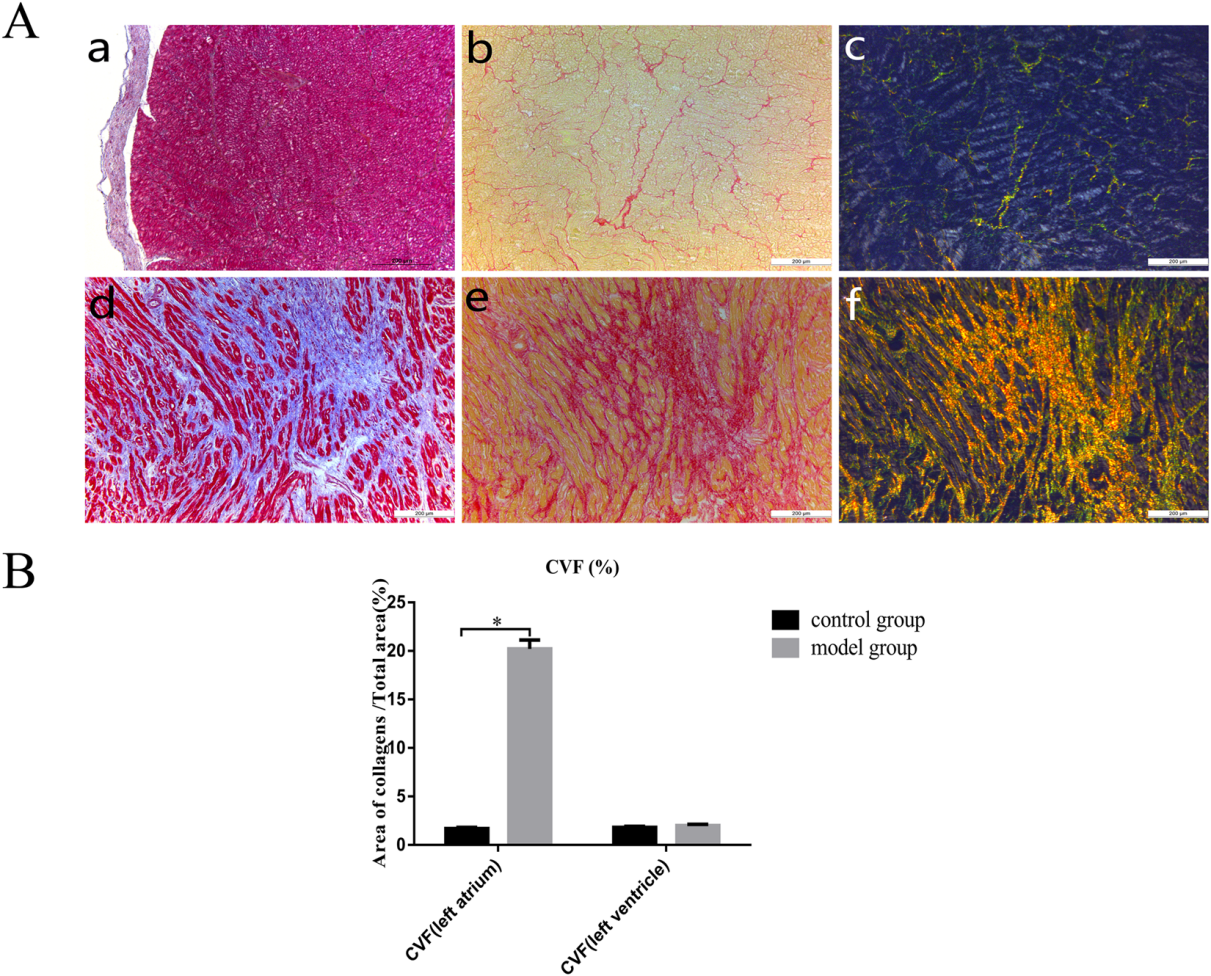
Histopathological evaluation of the structural remodelling of left atrial fibrosis. (**A**) Masson staining (a), Sirius Red staining (b) and polarized light microscopy (c) were performed in the normal group. Masson staining (d), Sirius Red staining (e) and polarized light microscopy (f) were performed in the model group. (**B**) The collagen volume fraction (CVF) was calculated as follows: CVF (%) = (total area of collagen/total area of the image) × 100%. *P < 0.05.

As shown in Fig. 1A, atrial tissue was stained by Masson’s trichrome and Sirius Red after sampling. Compared with the control group, the model group exhibited many collagen I and collagen III deposits, mainly in the myocardial interstitium, and a disordered arrangement. The CVF was 18.1 ± 3.0% in the model group and 1.6 ± 0.2% in the control group, and there was a significant difference between the two groups (P < 0.05). Compared with that of the left atrium, the CVF of the left ventricle in the model group was 2.0 ± 0.5% and was 1.9 ± 0.3% in the control group, and there was no significant difference between the two groups (P = 0.58) (Fig. 1B).

### Analysis of SIRT1 and collagen I protein expression by immunofluorescence in the left atrial tissue

**Figure 2:**
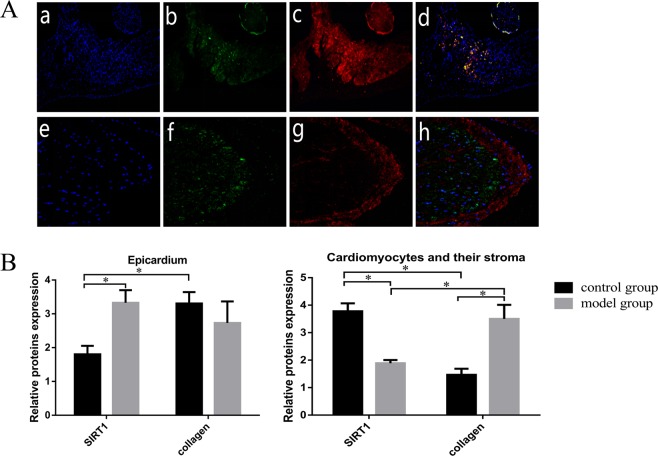
Immunofluorescence confocal microscopy showed the SIRT1 and collagen I protein expression profiles. The collagen I protein is represented by vimentin staining (red), the SIRT1 protein is represented green staining, and the nuclei are represented by DAPI staining (blue). (**A**) DAPI staining (a), SIRT1 (b), collagen I (c) and the merged figure (d) in the model group; DAPI staining (e), SIRT1 (f), collagen I (g) and the merged figure (h) in the control group. (**B**) Quantitative analysis of SIRT1 and collagen I expression in the cardiomyocytes and the epicardium. *P < 0.05.

As shown in Fig. 2A, the nuclei of the cardiomyocytes in each group were strongly stained. SIRT1 and collagen I were expressed in the myocardial cells and the interstitium and outer membrane.

Through immunofluorescence localization, we found that SIRT1 in the model group was distributed diffusely and mostly concentrated in the nuclei of the cardiomyocytes and myocardial adventitial tissue. Compared with that in the model group, SIRT1 in the control group was mostly concentrated in the cytoplasm and soma of the cardiomyocytes, and there was no SIRT1 expression in the tunica adventitia. Meanwhile, the deposition of collagen I was inconsistent with that of SIRT1. In the control group, compared with the model group, collagen I was mostly observed in the tunica adventitia. There was no deposition in the cardiomyocytes or their soma. An analysis of the localization of SIRT1 and collagen I showed differential distribution in the cardiomyocytes and their soma. SIRT1 aggregated in areas without collagen I deposition. The deposition of collagen I in areas in which SIRT1 was expressed was evidently decreased. Furthermore, we quantitatively analysed the expression of SIRT1 and collagen I by fluorescence detection. The results of the quantitative analysis were consistent with the fluorescence observations (Fig. 2B).

### Western blotting of SIRT1 and collagen I protein expression in the left atrial tissue

**Figure 3:**
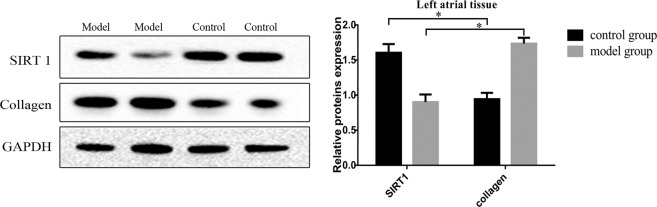
Western blotting for SIRT1 and collagen I protein expression in the left atrial tissue. The expression levels of collagen I and SIRT1 were detected via Western blotting with GAPDH as the normalization control. Cropped gels are used for presentation. Full-length blots are presented in Supplementary Figure 6–8. *P < 0.05.

The left atrial tissues were collected for Western blotting analysis. The samples were taken from areas with fibrosis. The results showed that the expression level of collagen I in the model group was higher than that in the control group and that the expression level of SIRT1 was significantly lower in the model group than in the control group (Fig. 3) (P < 0.05).

### Effect of resveratrol on the proliferation of NIH3T3 fibroblasts

**Figure 4:**
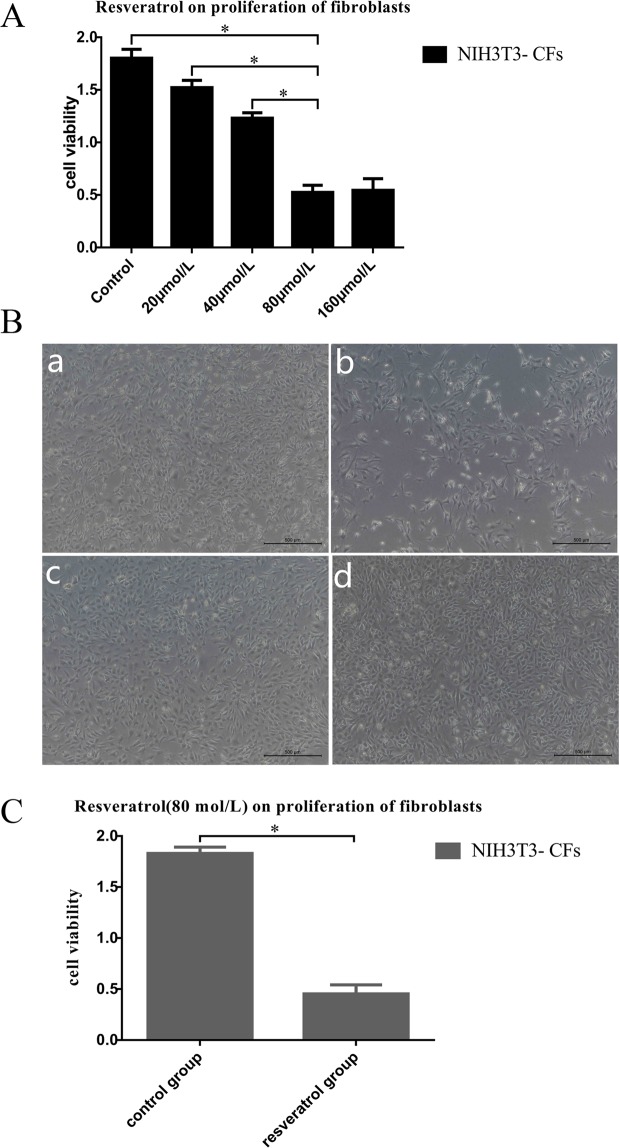
Effect of resveratrol on the proliferation of fibroblasts. (**A**) MTT analysis showed that the optimal concentration of resveratrol was 80 μmol/L. (**B**) The proliferation of the resveratrol group was significantly inhibited, and the degree of fibrosis was significantly decreased compared with that in the control group. (**C**) MTT analysis showed that cell viability decreased significantly in the resveratrol group compared to the control group. *P < 0.05.

In the early stage of the experiment, we applied different concentrations of resveratrol (0, 20, 40, 80 and 160 μmol/L) to NIH3T3 fibroblasts for 36 h, and the MTT method was used to detect cell activity. The optimal concentration of resveratrol was 80 μmol/L (Fig. 4A) (P < 0.05). That is, at 80 μmol/L resveratrol, the activity of the NIH3T3 myocardial fibroblasts showed the most significant inhibition.

Resveratrol (80 μmol/L) was selected to treat NIH3T3 cells for 36 h, and the proliferation of fibroblasts was observed under a microscope. As shown in Fig. 4B, the proliferation of the resveratrol group was significantly inhibited, and the degree of fibrosis was significantly decreased compared with that of the control group. MTT analysis showed that cell viability decreased significantly in the resveratrol group compared to the control group (Fig. 4C) (P < 0.05).

### Cell activity and protein expression in the NIH3T3 fibroblasts after treatment with Ang II

**Figure 5:**
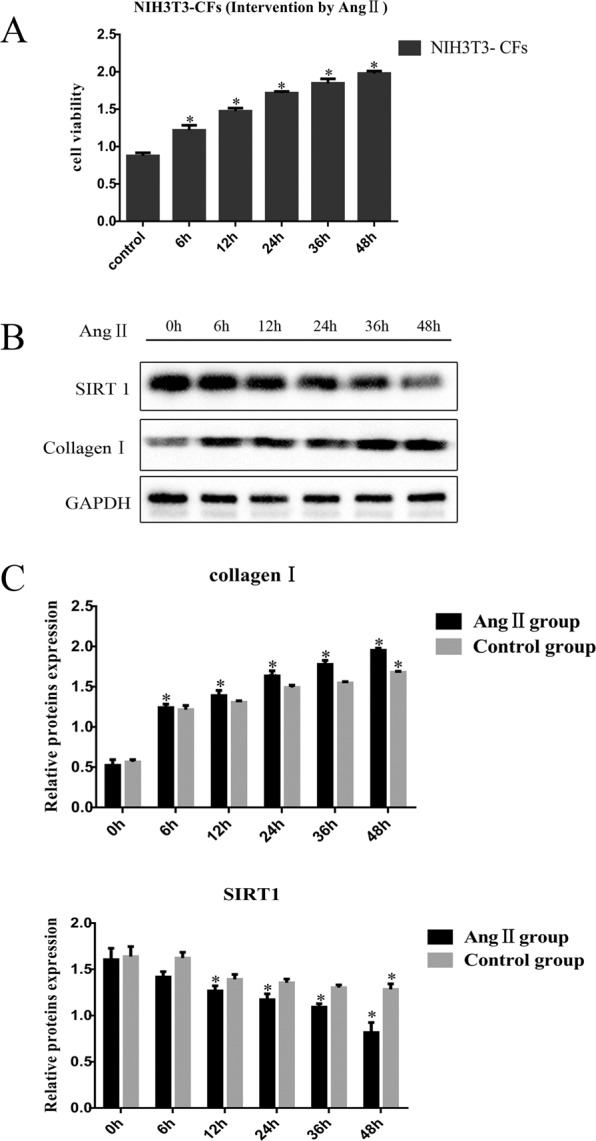
Cell activity and protein expression in the NIH3T3 fibroblasts after treatment with Ang II. (**A**) MTT analysis showed that cell viability increased gradually 0, 6, 12, 24, 36, and 48 h after treatment with Ang II. (**B** and **C**) Collagen I expression gradually increased 0, 6, 12, 24, 36, and 48 h after Ang II treatment, and SIRT1 expression gradually decreased, as determined by Western blotting. Cropped gels are used for presentation. Full-length blots are presented in Supplementary Figure 9–11. *P < 0.05.

After treatment with Ang II, MTT analysis showed that the cell viability increased significantly as the intervention time increased (Fig. 5A) (P < 0.05).

As shown in Fig. 5B,C in the NIH3T3 fibroblasts treated with Ang II, with the increase in time, the collagen I content gradually increased significantly. Furthermore, the content of SIRT1 decreased significantly. Overall, both the control group and the Ang II group showed different degrees of fibrosis; compared with that in the control group, the degree of fibrosis in the Ang II group was higher, and after treatment for 48 h, the content of collagen I in the Ang II group was significantly increased compared with that in the control group. Initially, there was no significant difference in the SIRT1 expression between the control group and the Ang II group. With the extension of culture time, the SIRT1 level in the Ang II group decreased significantly; the SIRT1 level in the control group showed a significant difference but only after 48 h after culture. Compared with that in the control group, the SIRT1 level in the Ang II group decreased significantly after culture for 12 h (P < 0.05).

## Discussion

In our study, we aimed to explore the regulatory effect of SIRT1 on the left atrial fibrosis induced by MR. We found differential distribution and expression of SIRT1 and collagen I in the left atrium, as determined by immunofluorescence and Western blotting. We also treated NIH3T3 fibroblasts (CFs) with resveratrol and Angiotensin II (Ang II) to analyse the specific mechanism involved in the development of myocardial fibrosis. In both the animal and cell experiments, the expression of collagen I in the MR group was increased significantly, along with SIRT1, compared to that in the control group. Therefore, SIRT1 might be a therapeutic target for preventing cardiac fibrosis induced by MR.

MR is a common disease in cardiac surgery. Patients often suffer from prolonged mitral regurgitation time, resulting in left atrial enlargement and atrial fibrotic remodelling. Clinically, mitral regurgitation is often associated with atrial fibrillation. Severe left atrial fibrosis is one of the fundamental causes of persistent atrial fibrillation. The occurrence of persistent atrial fibrillation is a challenging problem in clinical practice, and it is also a hot topic and difficult problem in research in this field.

SIRT1 (silent information regulator 1) is a histone deacetylase. This protein can sense the energy level in cells and delays cell senescence, helping cells resist external stress and improving metabolism^27,28^. SIRT1 has been confirmed by numerous experiments to participate in and regulate fibrosis in multiple organs and diseases. In 2005, Moynihan *et al*.^29^ was the first to successfully create a mouse model of high SIRT1 expression in islet beta cells through transgenic technology. This protein was shown to play an important role in liver fibrosis. In 2017, Zeng Z *et al*.^30^ found that the activation and overexpression of SIRT1 weakened pulmonary fibrosis through P300. In 2018, Li M *et al*.^31^ found that SIRT1 antagonizes liver fibrosis by blocking the activation of mouse hepatic stellate cells. In 2017, Ren Y *et al*.^32^ found that the SIRT1 activator SRT1720 alleviates renal fibrosis by inhibiting CTGF and oxidative stress.

In recent years, the number of studies on SIRT1 and the myocardium has also increased^33,34^. In 2016, Xiao J *et al*.^35^ found that curcumin protects against myocardial fibrosis caused by myocardial infarction by activating SIRT1. In 2017, Zhang J *et al*.^36^ found that astaxanthin reduces cardiac dysfunction and myocardial fibrosis caused by stress overload by activating SIRT1. Many studies have shown that SIRT1 is involved in the fibrotic process in various organs and diseases in the body, and the mechanism involved is relatively complex^37–39^. In 2017, Kim H *et al*.^40^ found that the antifibrotic effect of the Angiotensin II receptor blocker losartan is achieved by upregulating SIRT1 to inhibit endoplasmic reticulum stress and then inducing HO-1 and thioredoxin. In 2019, Li C^41^ found that the renin receptor Pro is involved in renal mitochondrial dysfunction, apoptosis and fibrosis in diabetic mice.

While our experiment was being conducted, several papers related to SIRT1 and myocardial fibrosis were published. In 2015, Chong E *et al*.^42^ found that resveratrol, a red wine antioxidant, reduces atrial fibrillation susceptibility in the failing heart through PI3K/AKT/Enos signalling pathway activation. In 2016, Cappetta D *et al*.^43^ found that SIRT1 activation attenuates diastolic dysfunction by reducing cardiac fibrosis in a model of anthracycline cardiomyopathy.

Based on previous studies and relevant experiments, we found that SIRT1 is closely related to myocardial fibrosis. Clinically, mitral regurgitation has a long observation period, and myocardial fibrosis caused by mitral regurgitation is also a gradually aggravated pathophysiological process. However, there is still a lack of experimental animal studies that fully simulate the clinical disease process and involved long-term observation. Moreover, no studies on SIRT1 and left atrial fibrosis caused by mitral regurgitation have been conducted previously.

In this study, we found that mitral regurgitation caused fibrotic remodelling, that the content of collagen I was increased significantly and that the content of SIRT1 was significantly reduced. By immunofluorescence localization, we found that there were differences in the SIRT1 and collagen I expression, and the results of the *in vitro* experiments are consistent with the animal studies. Thus, we found that SIRT1 expression was significantly decreased during the left atrial fibrosis induced by mitral regurgitation. The high expression of SIRT1 can significantly inhibit the proliferation of fibroblasts. SIRT1 plays an important role in the process of left atrial fibrosis caused by mitral regurgitation.

In our study, a large animal model of mitral regurgitation was used to simulate the course of clinical mitral regurgitation lesions. The initial aim of the trial was to simulate the clinical course of mitral regurgitation through long-term disease observation. The progressive enlargement of the left atrium through mitral regurgitation can lead to spontaneous atrial fibrillation or complications associated with heart failure. However, during the experiment, we found that progressive enlargement of the left atrium occurred in the model group but that the left ventricular function did not show significant abnormalities. No heart failure occurred in the animals in the model group, and the animals in the model group also did not exhibit spontaneous atrial fibrillation on the basis of left atrial enlargement. Therefore, in this experiment, large animal models of mitral regurgitation exhibited good homogeneity and stability, but it is difficult to build models of heart failure and atrial fibrillation through mitral regurgitation.

Sustained mitral regurgitation can lead to structural remodelling of the left atrium, with increased fibrosis and marked enlargement of the left atrium. In previous experiments, especially small animal experiments, it was difficult to simulate cardiac remodelling caused by valvular disease because small animal models are prone to complications such as heart failure and arrhythmia after modelling, and the models often died during the observation period. In this study, miniature pigs were used to construct a model of mitral regurgitation to study the regulation of SIRT1 on left atrial fibrosis caused by mitral regurgitation. The process of the clinical disease was largely recapitulated in the miniature pig model, and the preparation of large animal models ensured the homogeneity and stability of the experimental data, making the research more authentic and reliable. The regulation of left atrial fibrosis induced by mitral regurgitation by SIRT1 was demonstrated in both animal and cell experiments. In addition, to induce effective atrial fibrotic remodelling by mitral regurgitation, the observation period of the experimental animals was relatively long (30 months). Long-term observation and detection effectively simulated the process of atrial fibrotic remodelling caused by mitral regurgitation in clinical practice. The experimental results are convincing, and the conclusion is reliable.

## Conclusions

In both the animal experiments and the cell experiments, the expression of collagen I in the MR group was increased significantly compared to that in the control group, while the expression of SIRT1 was decreased. This study provides new ideas and methods for the diagnosis and treatment of left atrial fibrosis caused by mitral regurgitation and is expected to provide a new target for the prevention and treatment of atrial remodelling.

## Limitations and Strength

There are still many shortcomings in our research. First, observation and establishment of the animal models take a long time. During the process of animal modelling, many SIRT1-related articles were published. Many previous studies have reported the link between SIRT1 and fibrosis in recent years in multiple organs, so the innovation of our article has been substantially reduced. Second, the mechanism of atrial fibrosis induced by mitral regurgitation is complicated. The increase in Angiotensin II is one of the factors that aggravates atrial fibrosis^44^, but it is by no means the only mechanism. Therefore, the pathophysiological process of atrial fibrosis caused by mitral regurgitation cannot be completely simulated *in vitro* experiments. In addition, with the development of cardiac-specific fibroblasts in recent years, the use of cardiac-specific fibroblasts would strengthen the link between *in vitro* and *in vivo* experiments. Finally, the mechanism and influence of SIRT1 on atrial fibrosis caused by mitral regurgitation needs to be explored and verified. By artificially altering SIRT1 expression or activity *in vivo*, it should be possible to prevent or partially rescue profibrotic conditions induced by MR. This hypothesis will be further explored in later experiments.

Mitral regurgitation-induced left atrial fibrosis is a chronic disease process. In previous experimental studies, there were few studies on mitral regurgitation because the mitral regurgitation model was difficult to establish. Even if the mitral regurgitation model was established, the observation time of the model was short due to complications and high mortality. In the literature, our study reported the longest animal experimental model, and the model has good homogeneity and stability and can drastically simulate clinical mitral regurgitation leading to the pathological process of left atrial fibrosis. These results may contribute to the treatment of mitral valve regurgitation caused by left atrial fibrosis and targeting SIRT1 may be a treatment strategy for mitral regurgitation and left atrial fibrosis.

## Supplementary information

Supporting Information.

## References

[1] Jalife J, Kaur K (2015). Atrial remodeling, fibrosis, and atrial fibrillation. Trends Cardiovasc Med.

[2] Vasquez N (2019). Low Left Atrial Strain Is Associated With Adverse Outcomes in Hypertrophic Cardiomyopathy Patients. J Am Soc Echocardiogr.

[3] Kamphuis VP (2019). Electrical remodeling after percutaneous atrial septal defect closure in pediatric and adult patients. Int J Cardiol.

[4] Thomas L, Abhayaratna WP (2017). Left Atrial Reverse Remodeling: Mechanisms, Evaluation, and Clinical Significance. JACC Cardiovasc Imaging.

[5] Honigberg MC (2019). Electrocardiographic findings in peripartum cardiomyopathy. Clin Cardiol.

[6] Agoston-Coldea L, Lupu S, Hicea S, Mocan T (2013). Left atrium systolic and diastolic function assessment in hypertensive patients with preserved ejection fraction. Acta Physiol Hung.

[7] Kainuma S (2011). Advanced left-atrial fibrosis is associated with unsuccessful maze operation for valvular atrial fibrillation. Eur J Cardiothorac Surg.

[8] McManus DD, Shaikh AY, Abhishek F, Vasan RS (2011). Atrial fibrillation and heart failure parallels: lessons for atrial fibrillation prevention. Crit Pathw Cardiol.

[9] Xiao P (2011). Blockade of angiotensin II improves hyperthyroid induced abnormal atrial electrophysiological properties. Regul Pept.

[10] Li GL (2019). Atrial dysplasia in the atria of humans without cardiovascular disease. J Investig Med.

[11] den Uijl DW (2011). Impact of left atrial fibrosis and left atrial size on the outcome of catheter ablation for atrial fibrillation. Heart.

[12] Gibson DN (2011). Stiff left atrial syndrome after catheter ablation for atrial fibrillation: clinical characterization, prevalence, and predictors. Heart Rhythm.

[13] Laurent G (2013). Permanent left atrial pacing therapy may improve symptoms in heart failure patients with preserved ejection fraction and atrial dyssynchrony: a pilot study prior to a national clinical research programme. Eur J Heart Fail.

[14] Li XZ, Cheng LZ, Yan YM, Liu BH, Cheng YX (2019). SIRT1 inhibitory compounds from the roots of Codonopsis pilosula. J Asian Nat Prod Res.

[15] Yang XW, Ma LY, Zhou QL, Xu W, Zhang YB (2018). SIRT1 activator isolated from artificial gastric juice incubate of total saponins in stems and leaves of Panax ginseng. Bioorg Med Chem Lett.

[16] Wan X, Ahmad H, Zhang L, Wang Z, Wang T (2018). Dietary enzymatically treated Artemisia annua L. improves meat quality, antioxidant capacity and energy status of breast muscle in heat-stressed broilers. J Sci Food Agric.

[17] Asghari S, Asghari-Jafarabadi M, Somi MH, Ghavami SM, Rafraf M (2018). Comparison of Calorie-Restricted Diet and Resveratrol Supplementation on Anthropometric Indices, Metabolic Parameters, and Serum Sirtuin-1 Levels in Patients With Nonalcoholic Fatty Liver Disease: A Randomized Controlled Clinical Trial. J Am Coll Nutr.

[18] Hwang JS (2018). Formononetin inhibits lipopolysaccharide-induced release of high mobility group box 1 by upregulating SIRT1 in a PPARδ-dependent manner. PeerJ.

[19] Mishra M, Duraisamy AJ, Kowluru RA (2018). Sirt1: A Guardian of the Development of Diabetic Retinopathy. Diabetes.

[20] Tanno M, Kuno A, Horio Y, Miura T (2012). Emerging beneficial roles of sirtuins in heart failure. Basic Res Cardiol.

[21] Vinciguerra M (2012). mIGF-1/JNK1/SirT1 signaling confers protection against oxidative stress in the heart. Aging Cell.

[22] Xing, T., *et al* Raspberry Supplementation Improves Insulin Signaling and Promotes Brown-Like Adipocyte Development in White Adipose Tissue of Obese Mice. *Mol Nutr Food Res* **62** (2018). 10.1002/mnfr.201701035 29322691

[23] Saidi D (2018). Glioma-induced SIRT1-dependent activation of hMOF histone H4 lysine 16 acetyltransferase in microglia promotes a tumor supporting phenotype. Oncoimmunology.

[24] Li B (2018). The characteristics of a porcine mitral regurgitation model. Exp Anim.

[25] Cui YC (2014). A pig model of ischemic mitral regurgitation induced by mitral chordae tendinae rupture and implantation of an ameroid constrictor. PLoS One.

[26] Gao Y, Chu M, Hong J, Shang J, Xu D (2014). Hypoxia induces cardiac fibroblast proliferation and phenotypic switch: a role for caveolae and caveolin-1/PTEN mediated pathway. J Thorac Dis.

[27] Chen Y (2017). Endogenous Nampt upregulation is associated with diabetic nephropathy inflammatory-fibrosis through the NF-κB p65 and Sirt1 pathway; NMN alleviates diabetic nephropathy inflammatory-fibrosis by inhibiting endogenous Nampt. Exp Ther Med.

[28] Abd EMDM, IAAE I, Elshazly SM (2017). Sildenafil protects against bile duct ligation induced hepatic fibrosis in rats: Potential role for silent information regulator 1 (SIRT1). Toxicol Appl Pharmacol.

[29] Moynihan KA (2005). Increased dosage of mammalian Sir2 in pancreatic beta cells enhances glucose-stimulated insulin secretion in mice. Cell Metab.

[30] Zeng Z (2017). Activation and overexpression of Sirt1 attenuates lung fibrosis via P300. Biochem Biophys Res Commun.

[31] Li M (2018). SIRT1 antagonizes liver fibrosis by blocking hepatic stellate cell activation in mice. FASEB J.

[32] Ren Y (2017). The Sirt1 activator, SRT1720, attenuates renal fibrosis by inhibiting CTGF and oxidative stress. Int J Mol Med.

[33] Cappetta D (2016). SIRT1 activation attenuates diastolic dysfunction by reducing cardiac fibrosis in a model of anthracycline cardiomyopathy. Int J Cardiol.

[34] Chong E (2015). Resveratrol, a red wine antioxidant, reduces atrial fibrillation susceptibility in the failing heart by PI3K/AKT/eNOS signaling pathway activation. Heart Rhythm.

[35] Xiao J, Sheng X, Zhang X, Guo M, Ji X (2016). Curcumin protects against myocardial infarction-induced cardiac fibrosis via SIRT1 activation *in vivo* and *in vitro*. Drug Des Devel Ther.

[36] Zhang J (2017). Astaxanthin attenuated pressure overload-induced cardiac dysfunction and myocardial fibrosis: Partially by activating SIRT1. Biochim Biophys Acta Gen Subj.

[37] Han B (2019). Dietary melatonin attenuates chromium-induced lung injury via activating the Sirt1/Pgc-1α/Nrf2 pathway. Food Funct.

[38] Lo CS (2017). Heterogeneous Nuclear Ribonucleoprotein F Stimulates Sirtuin-1 Gene Expression and Attenuates Nephropathy Progression in Diabetic Mice. Diabetes.

[39] Li K (2019). Tetrahydrocurcumin Ameliorates Diabetic Cardiomyopathy by Attenuating High Glucose-Induced Oxidative Stress and Fibrosis via Activating the SIRT1 Pathway. Oxid Med Cell Longev.

[40] Kim, H. et al. Anti-Fibrotic Effect of Losartan, an Angiotensin II Receptor Blocker, Is Mediated through Inhibition of ER Stressvia Up-Regulation of SIRT1, Followed by Induction of HO-1 and Thioredoxin. *Int J Mol Sci* **18** (2017). 10.3390/ijms18020305 PMC5343841 28146117

[41] Li C, Matavelli LC, Akhtar S, Siragy HM (2019). Pro)renin receptor contributes to renal mitochondria dysfunction, apoptosis and fibrosis in diabetic mice. Sci Rep.

[42] Chong E (2015). Resveratrol, a red wine antioxidant, reduces atrial fibrillation susceptibility in the failing heart by PI3K/AKT/eNOS signaling pathway activation. Heart Rhythm.

[43] Cappetta D (2016). SIRT1 activation attenuates diastolic dysfunction by reducing cardiac fibrosis in a model of anthracycline cardiomyopathy. Int J Cardiol.

[44] Kang DH (2019). Angiotensin Receptor Neprilysin Inhibitor for Functional Mitral Regurgitation. Circulation.

