# Supplementary material for: Regulation of left atrial fibrosis induced by mitral regurgitation by SIRT1
Source: Sci Rep. 2020 Apr 29;10:7278. doi: 10.1038/s41598-020-64308-6 (PMC7190846; doi:10.1038/s41598-020-64308-6)
Supplement: Supplementary file 1 — Supporting Information. [file 41598_2020_64308_MOESM1_ESM.pdf]

Regulation of left atrial fibrosis induced by mitral regurgitation by SIRT1

Dong Zhang[1], Bo Li[2], Bin Li[3], Yue Tang[3*]

[1](Beijing JiShuiTan Hospital, Department of Thoracic Surgery, Beijing, China), [2](The Seventh Affiliated Hospital, Sun Yat-sen University, Department of Cardiac Surgery, Shenzhen, China), [3](Animal Experimental Centre, Beijing Key Laboratory of Preclinical Research and Evaluation for Cardiovascular Implant Materials, State Key Laboratory of Cardiovascular Disease, Fuwai Hospital, National Centre for Cardiovascular Disease, Chinese Academy of Medical Sciences and Peking Union Medical College, Beijing, China)

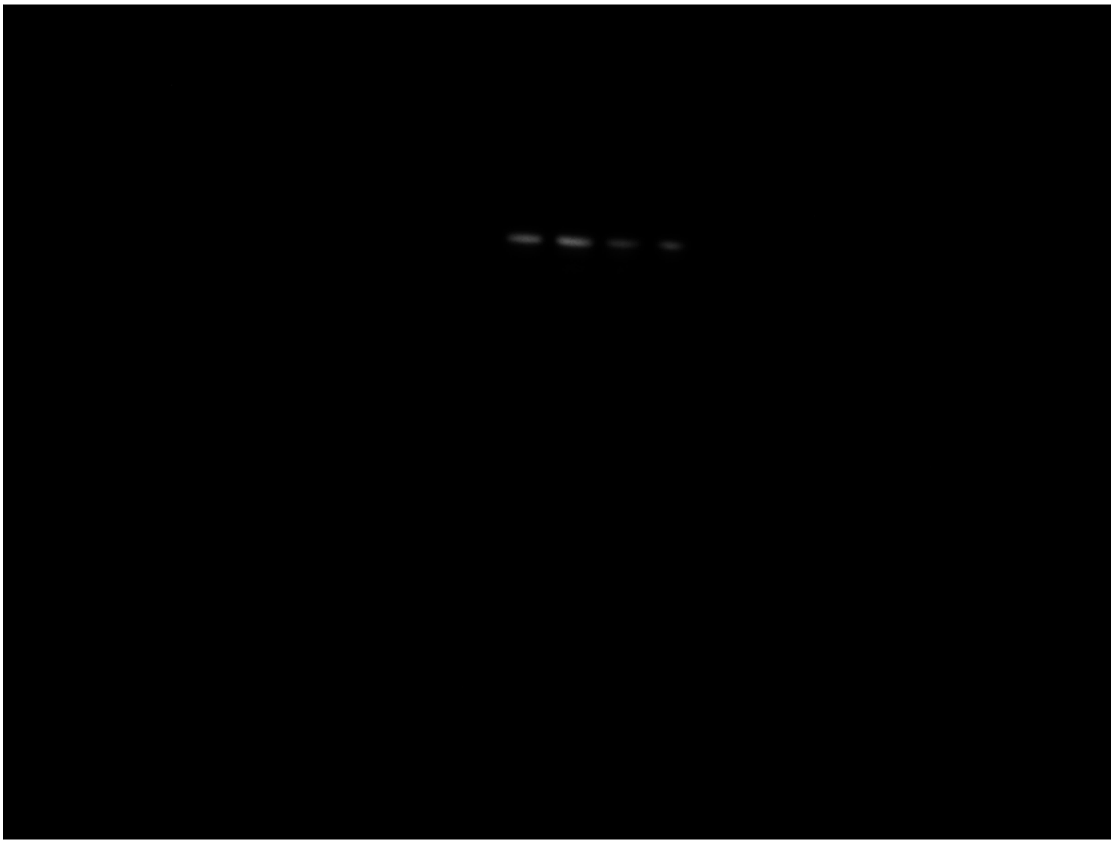

Figure 6A. Full-length blot: Multiple exposures of Figure3-Collagen is presented.

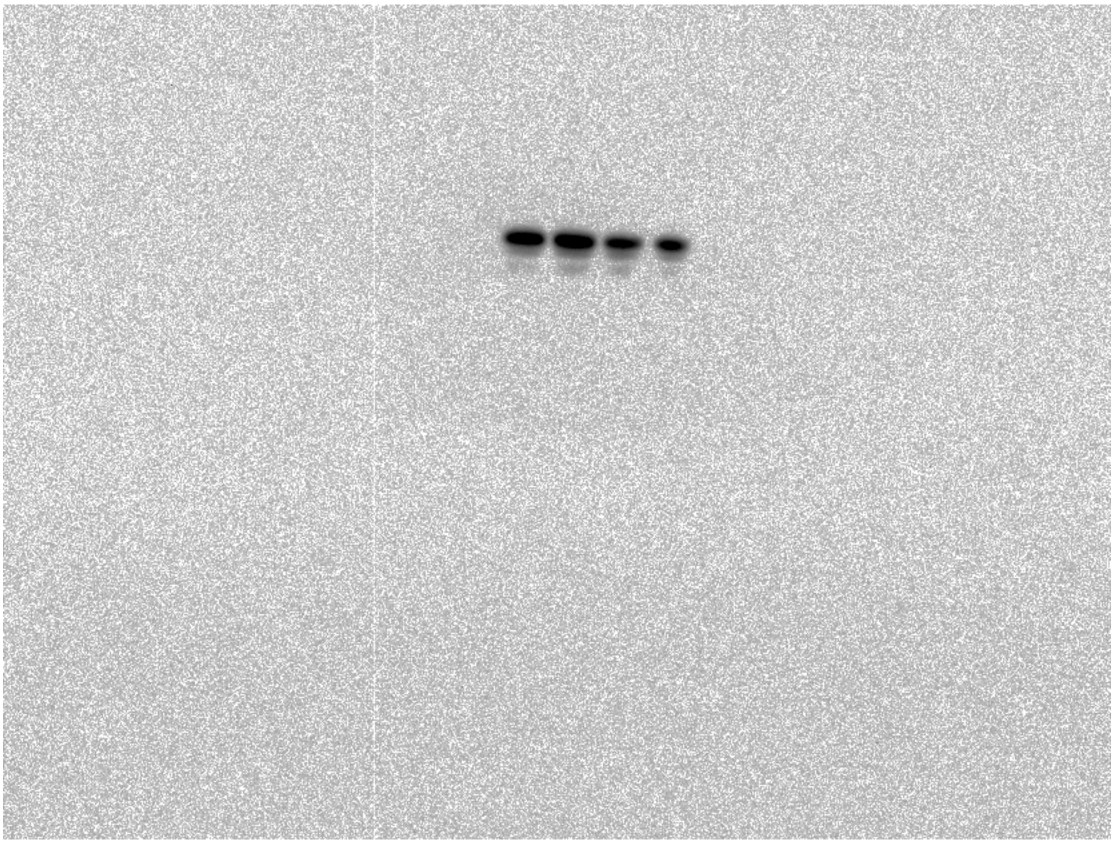

Figure 6B. Full-length blot: Multiple exposures of Figure3-Collagen is presented.

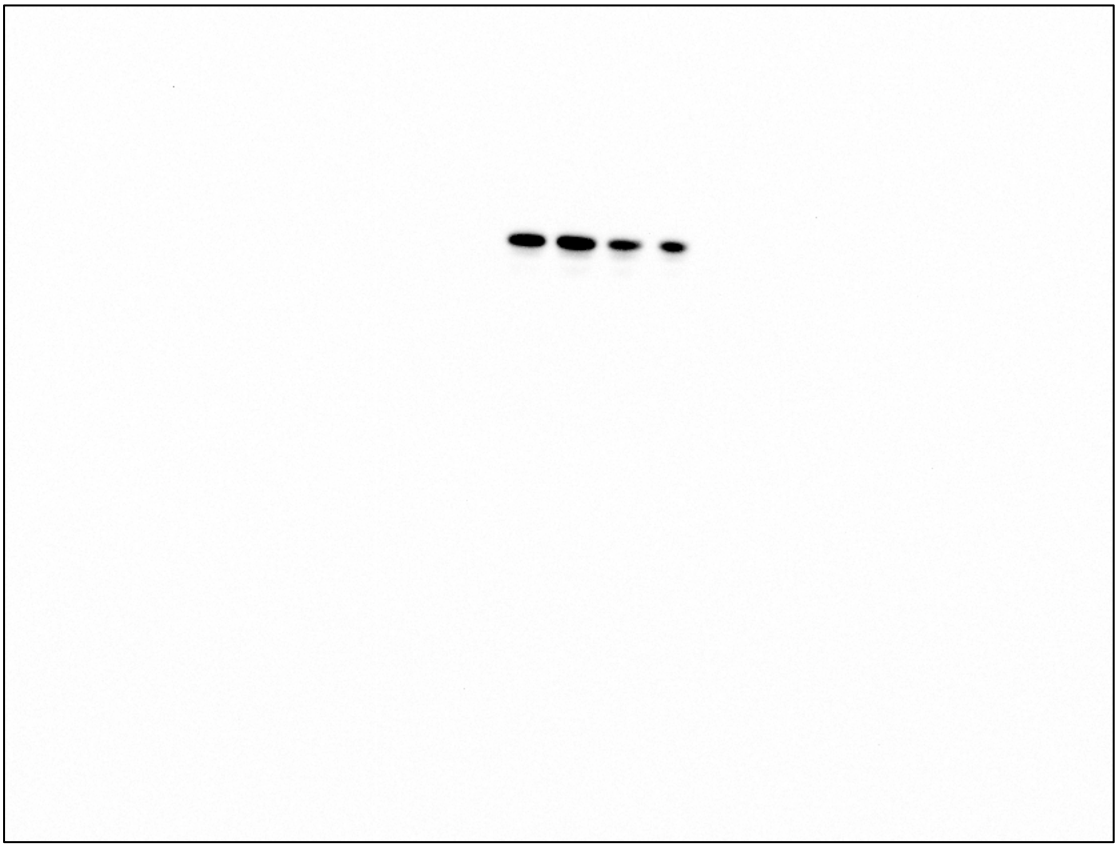

Figure 6C. Full-length blot: Multiple exposures of Figure3-Collagen is presented.

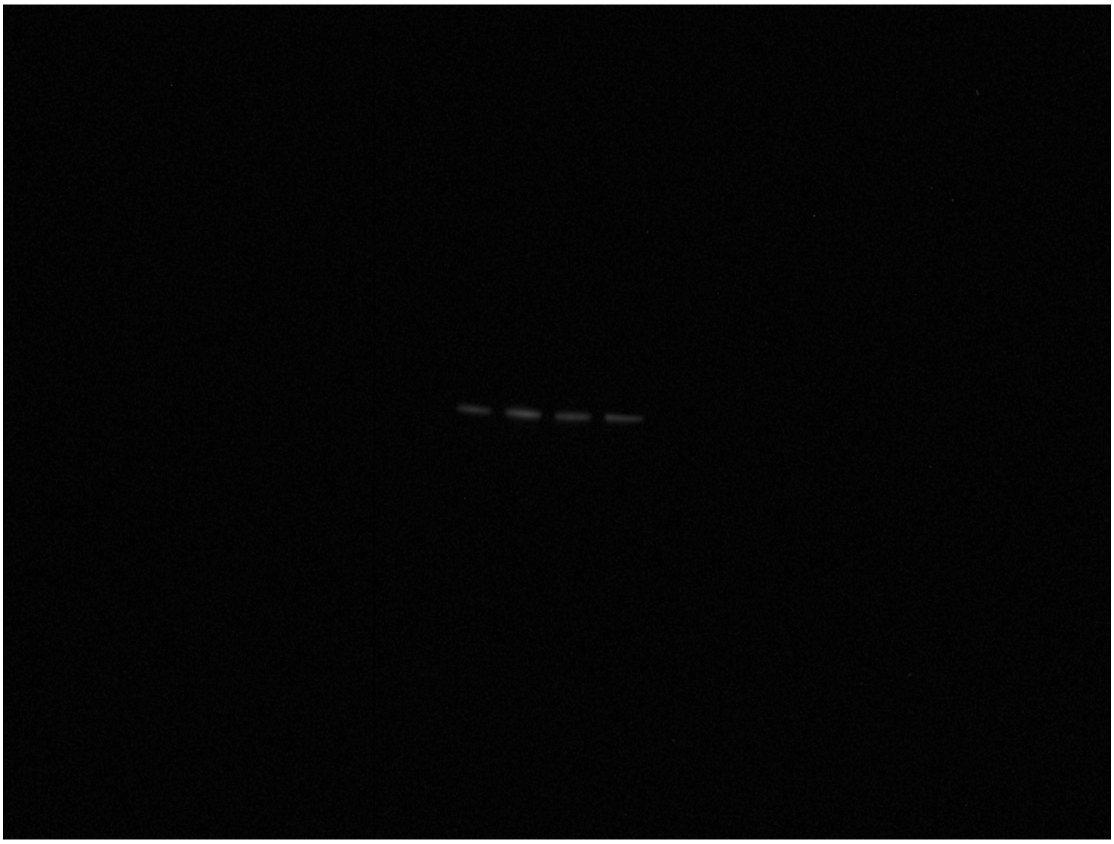

Figure 7A. Full-length blot: Multiple exposures of Figure3-GAPDH is presented.

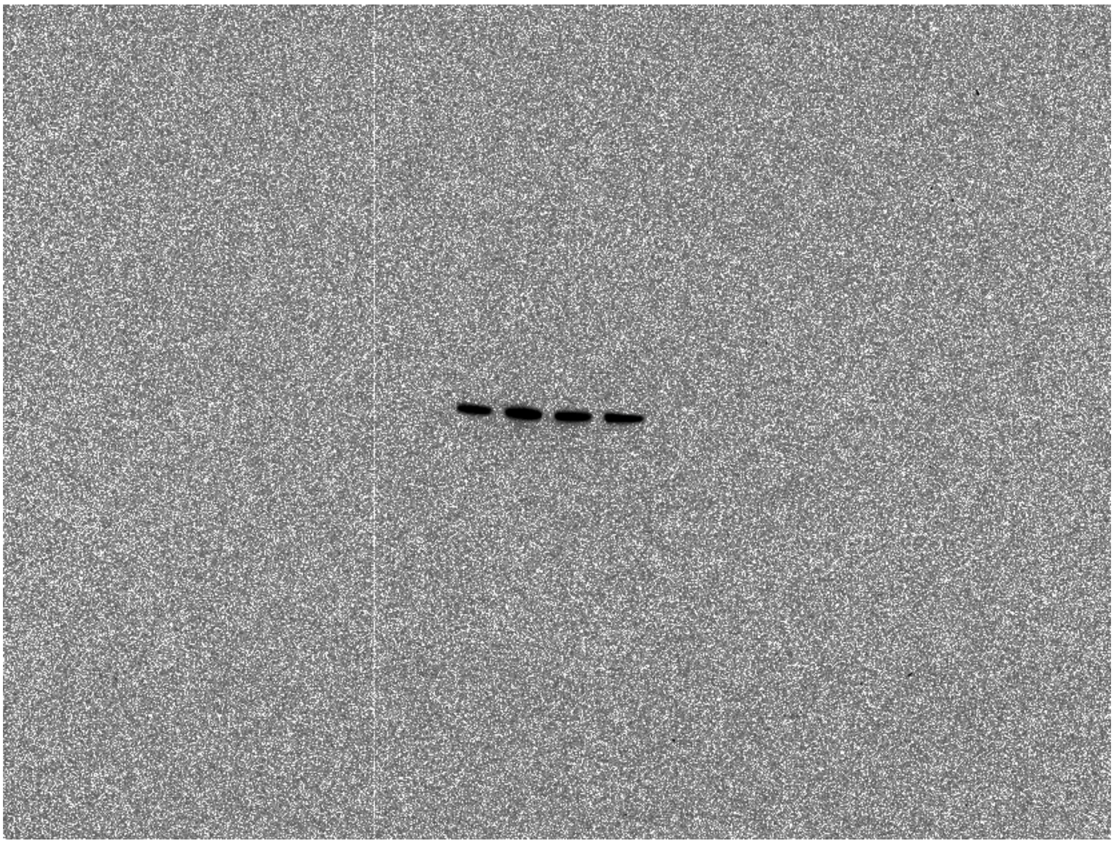

Figure 7B. Full-length blot: Multiple exposures of Figure3-GAPDH is presented.

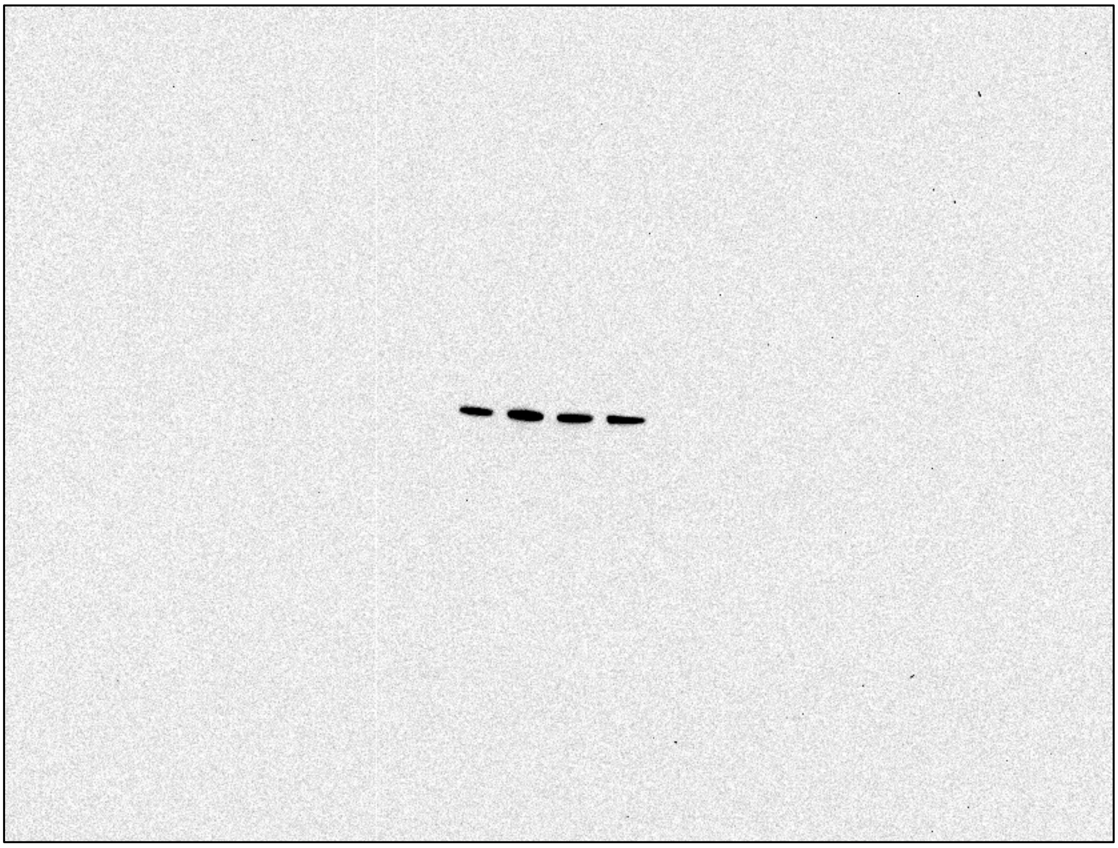

Figure 7C. Full-length blot: Multiple exposures of Figure3-GAPDH is presented.

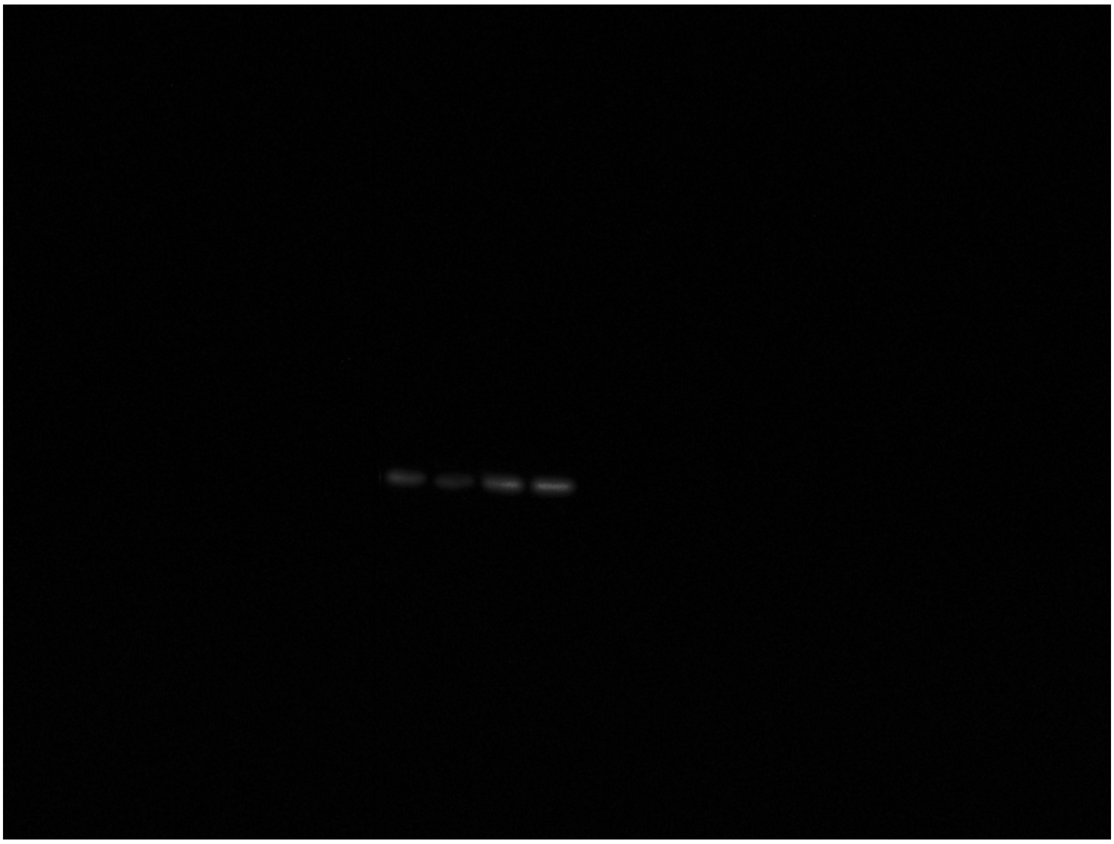

Figure 8A. Full-length blot: Multiple exposures of Figure3-SIRT1 is presented.

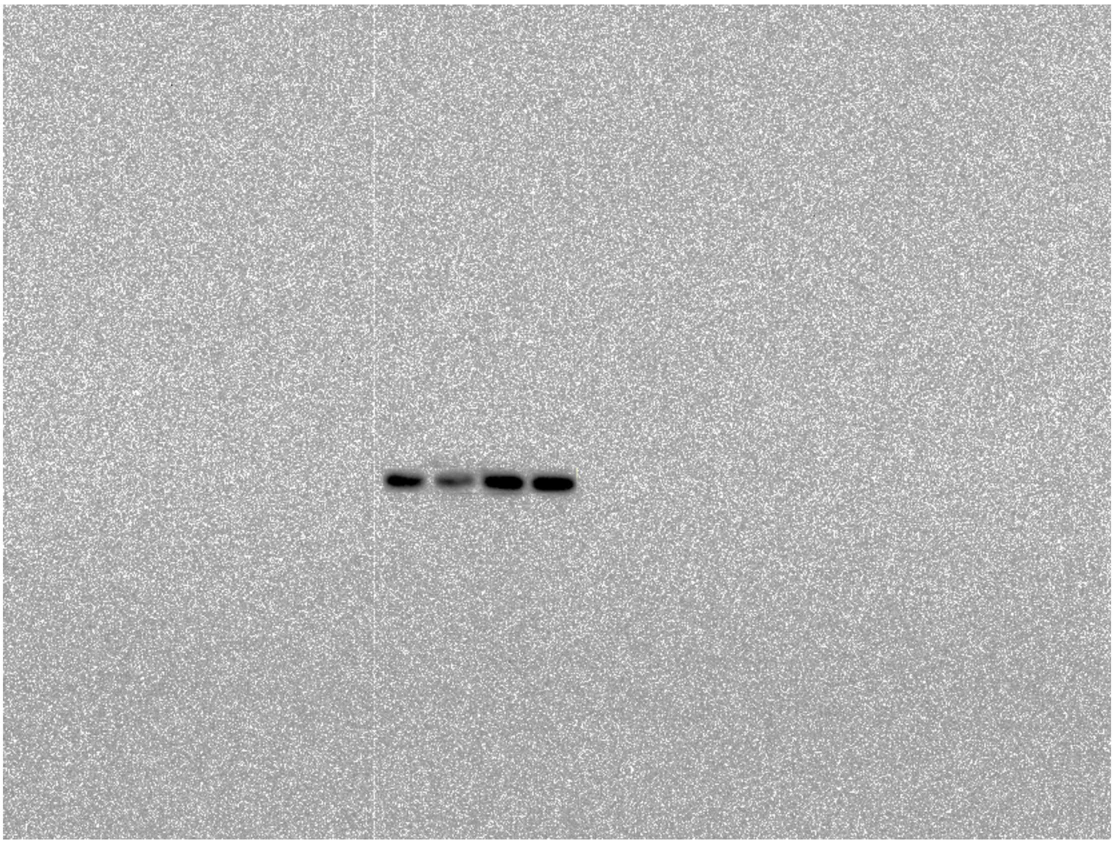

Figure 8B. Full-length blot: Multiple exposures of Figure3-SIRT1 is presented.

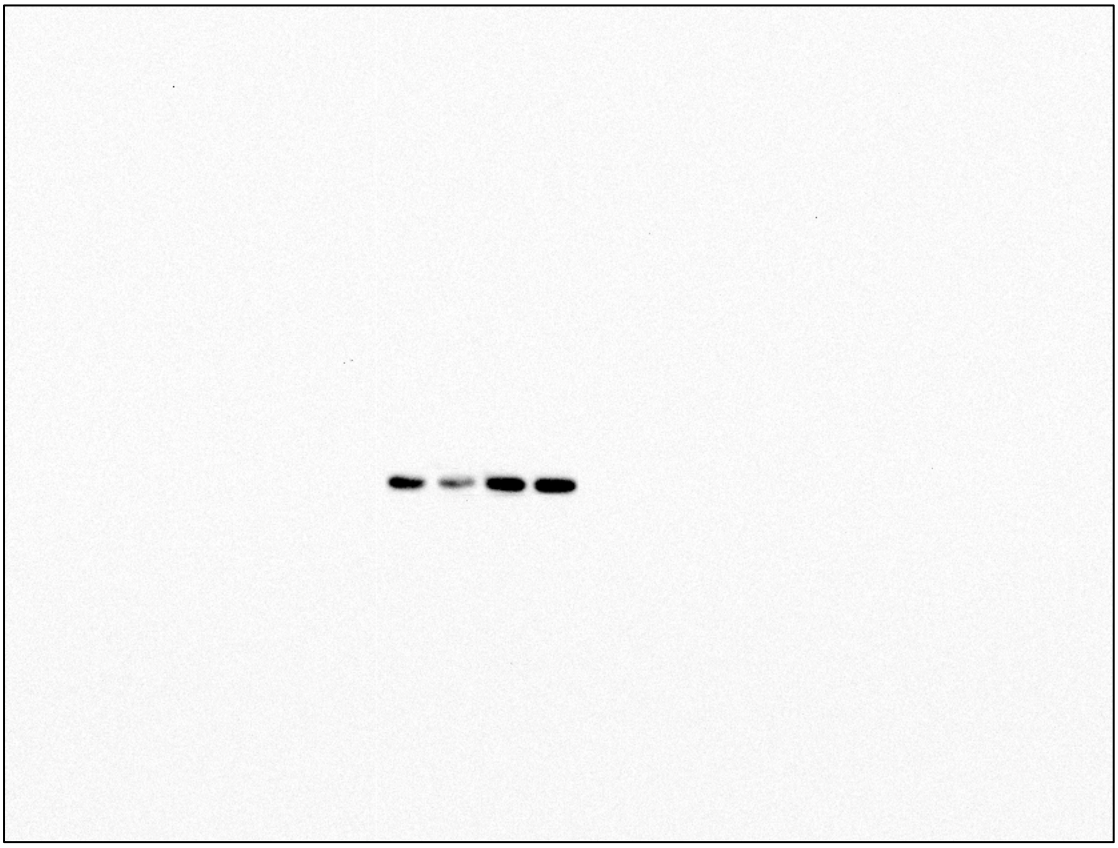

Figure 8C. Full-length blot: Multiple exposures of Figure3-SIRT1 is presented.

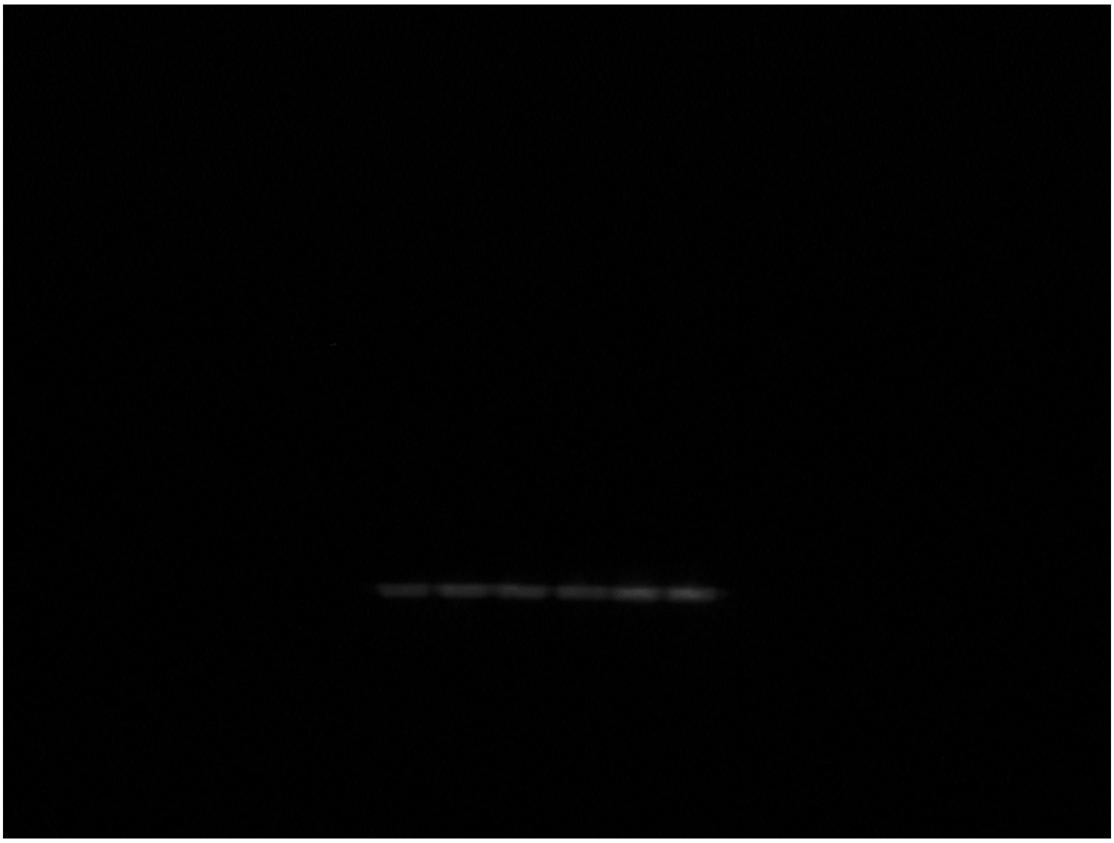

Figure 9A. Full-length blot: Multiple exposures of Figure5-Collagen is presented.

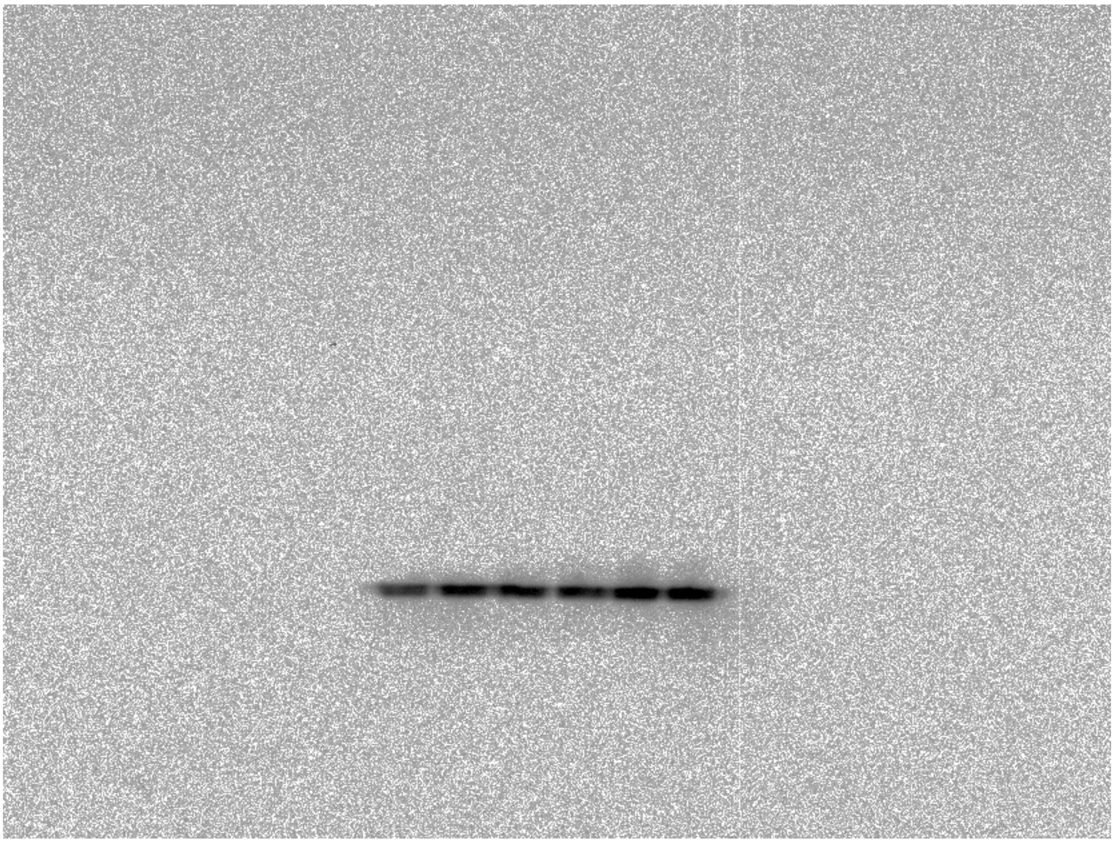

Figure 9B. Full-length blot: Multiple exposures of Figure5-Collagen is presented.

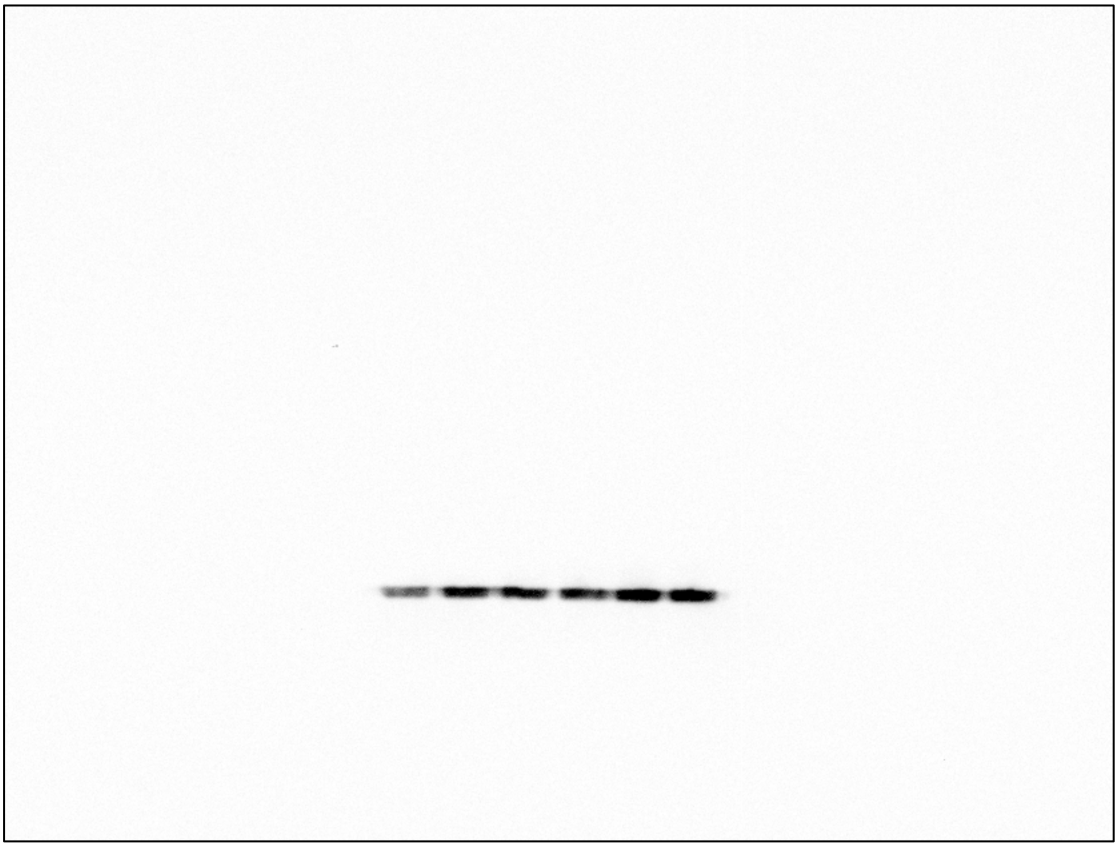

Figure 9C. Full-length blot: Multiple exposures of Figure5-Collagen is presented.

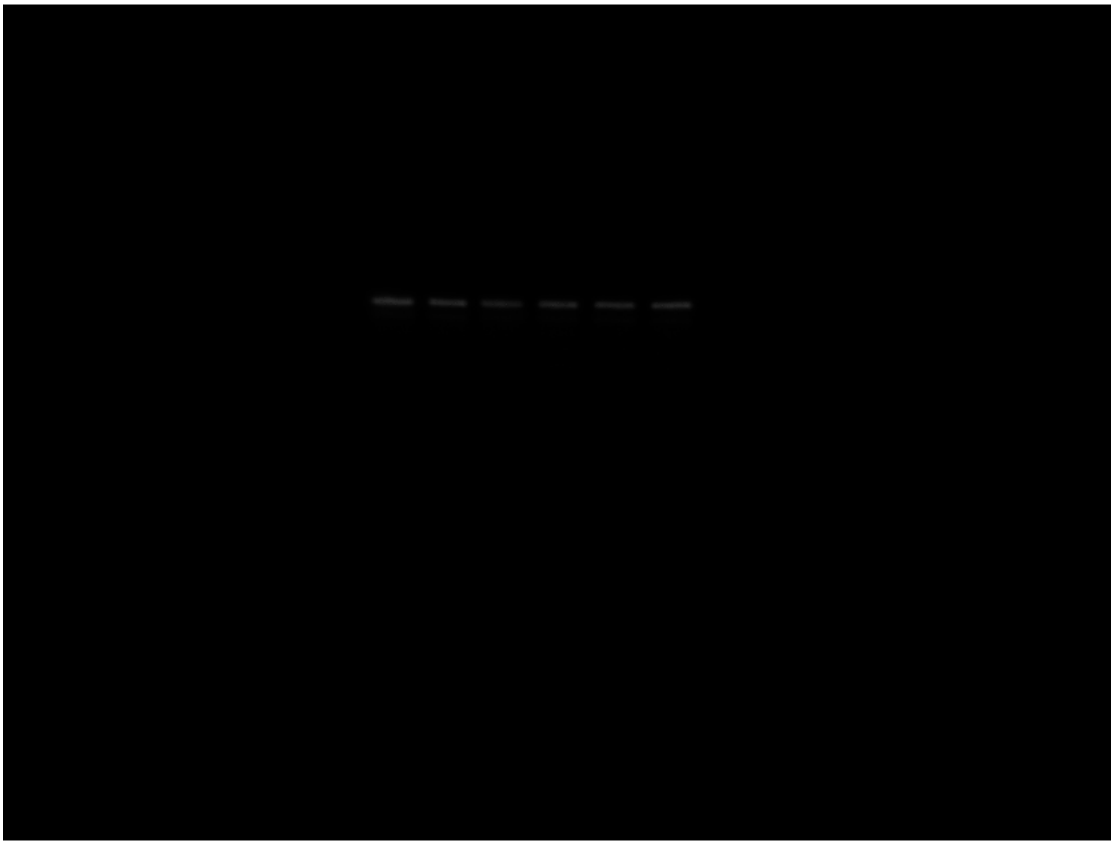

Figure10A. Full-length blot: Multiple exposures of Figure5-GAPDH is presented.

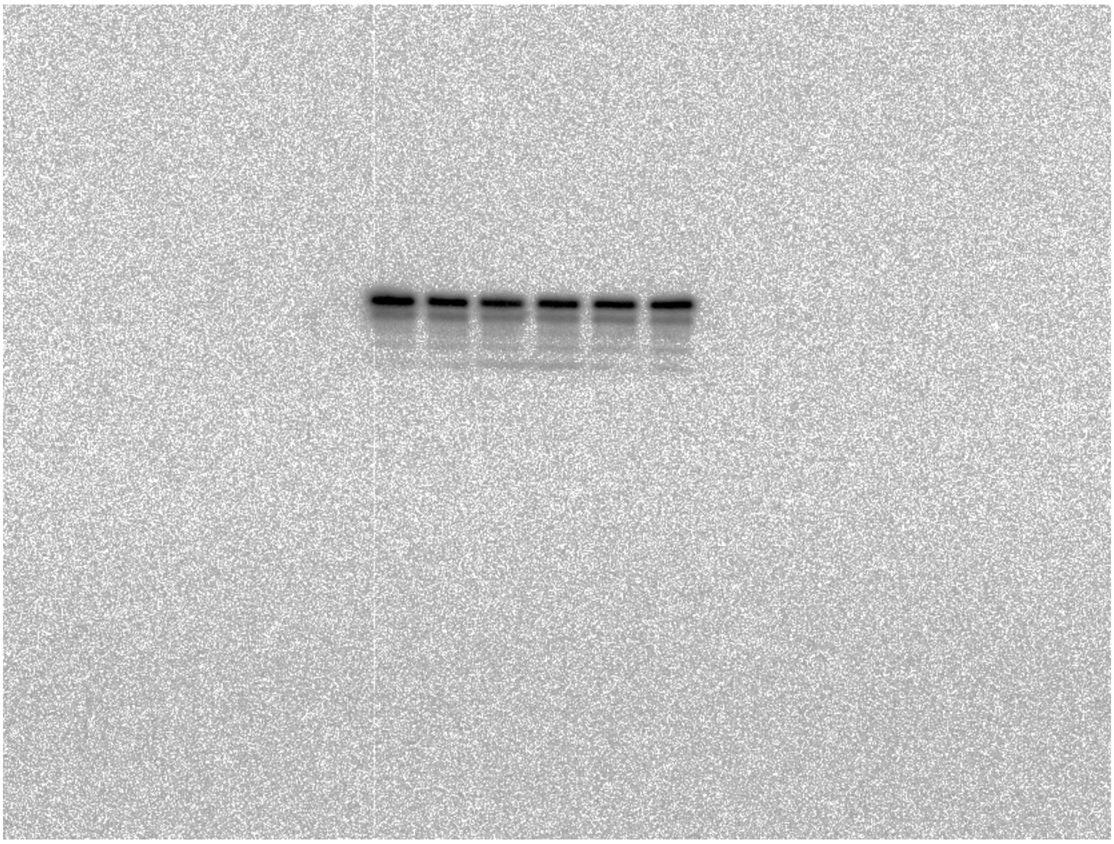

Figure10B. Full-length blot: Multiple exposures of Figure5-GAPDH is presented.

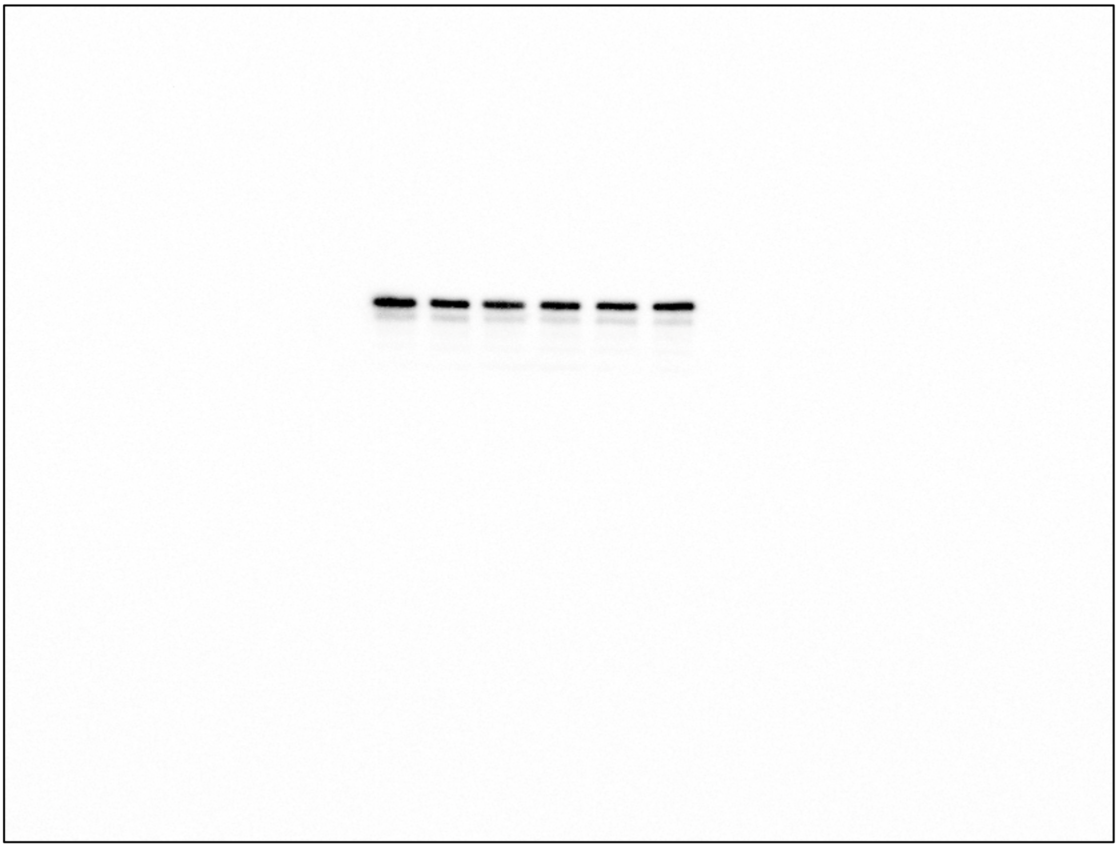

Figure10C. Full-length blot: Multiple exposures of Figure5-GAPDH is presented.

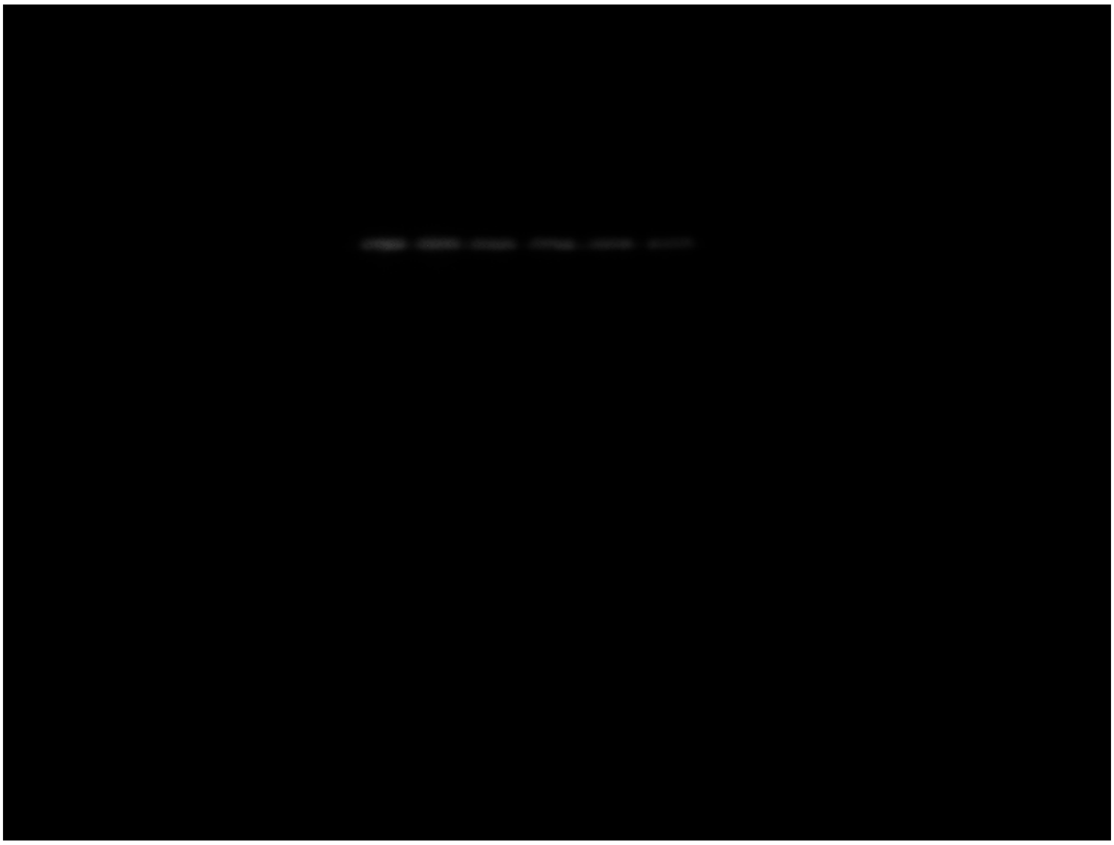

Figure11A. Full-length blot: Multiple exposures of Figure5-SIRT1 is presented.

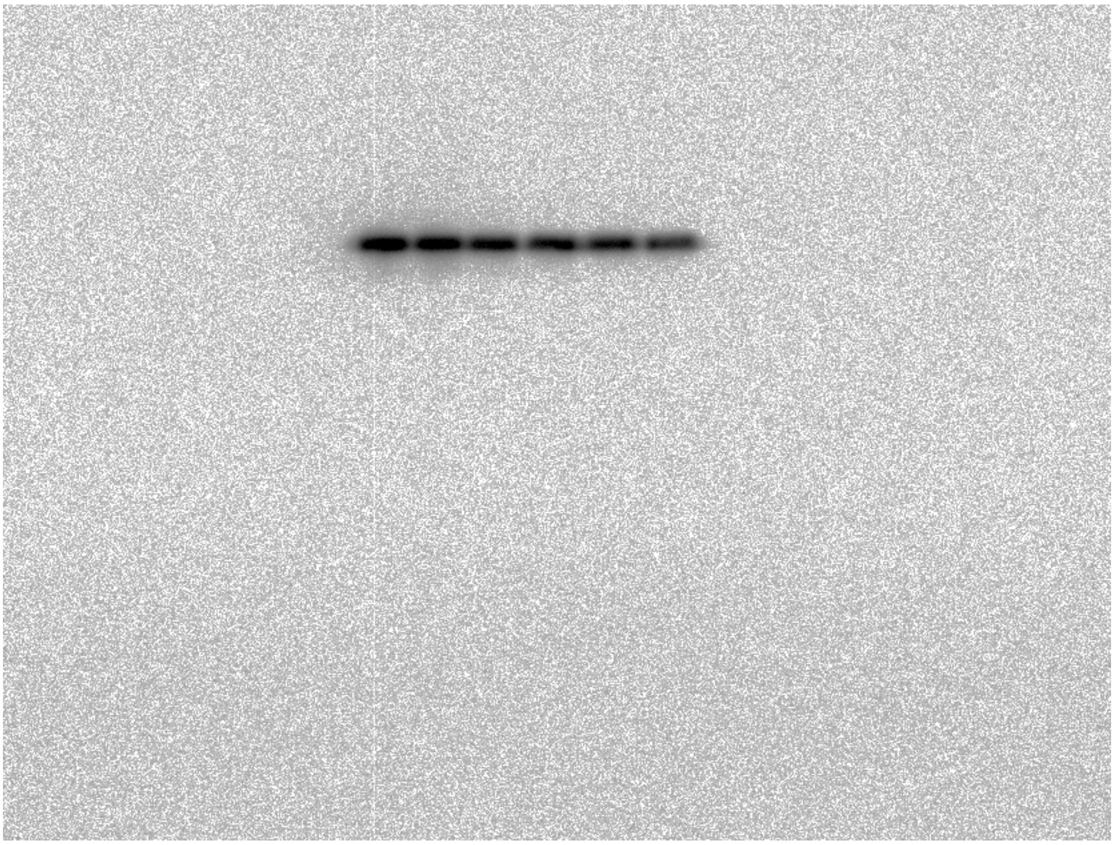

Figure11B. Full-length blot: Multiple exposures of Figure5-SIRT1 is presented.

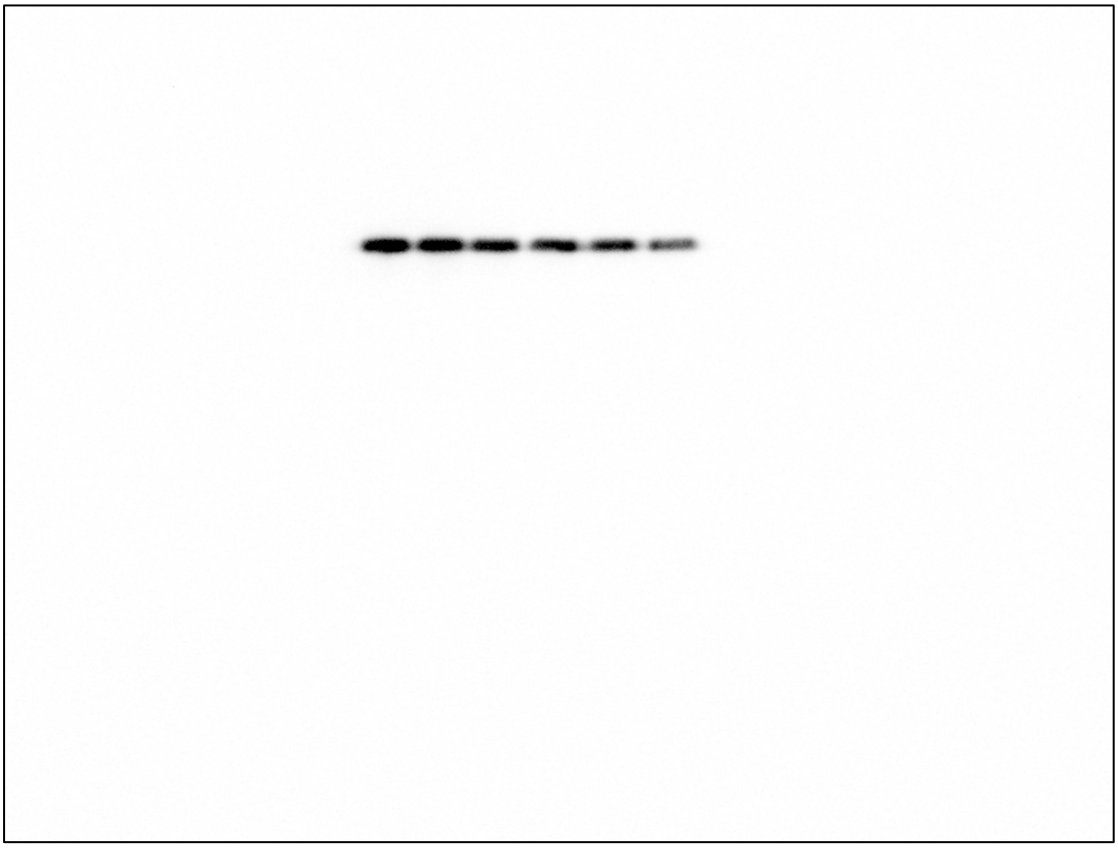

Figure11C. Full-length blot: Multiple exposures of Figure5-SIRT1 is presented.
